# Exogenous application of xanthine and uric acid and nucleobase-ascorbate transporter MdNAT7 expression regulate salinity tolerance in apple

**DOI:** 10.1186/s12870-021-02831-y

**Published:** 2021-01-19

**Authors:** Tingting Sun, Tingting Pei, Lulu Yang, Zhijun Zhang, Mingjun Li, Yuerong Liu, Fengwang Ma, Changhai Liu

**Affiliations:** 1 State Key Laboratory of Crop Stress Biology for Arid Areas/Shaanxi Key Laboratory of Apple, College of Horticulture, Northwest A&F University, Yangling, 712100 Shaanxi China; 2 Beijing Academy of Forestry and Pomology Sciences, Beijing Engineering Research Center for Deciduous Fruit Trees, Key Laboratory of Biology and Genetic Improvement of Horticultural Crops (North China), Ministry of Agriculture and Rural Affairs, Beijing, 100093 People’s Republic of China

**Keywords:** Apple, Antioxidant system, Nucleobase-ascorbate transporter, Salinity stress, Uric acid, Xanthine

## Abstract

**Background:**

Soil salinity is a critical threat to global agriculture. In plants, the accumulation of xanthine activates xanthine dehydrogenase (XDH), which catalyses the oxidation/conversion of xanthine to uric acid to remove excess reactive oxygen species (ROS). The nucleobase-ascorbate transporter (NAT) family is also known as the nucleobase-cation symporter (NCS) or AzgA-like family. NAT is known to transport xanthine and uric acid in plants. The expression of *MdNAT* is influenced by salinity stress in apple.

**Results:**

In this study, we discovered that exogenous application of xanthine and uric acid enhanced the resistance of apple plants to salinity stress. In addition, *MdNAT7* overexpression transgenic apple plants showed enhanced xanthine and uric acid concentrations and improved tolerance to salinity stress compared with nontransgenic plants, while opposite phenotypes were observed for *MdNAT7* RNAi plants. These differences were probably due to the enhancement or impairment of ROS scavenging and ion homeostasis abilities.

**Conclusion:**

Our results demonstrate that xanthine and uric acid have potential uses in salt stress alleviation, and *MdNAT7* can be utilized as a candidate gene to engineer resistance to salt stress in plants.

**Supplementary Information:**

The online version contains supplementary material available at 10.1186/s12870-021-02831-y.

## Background

Soil salinity is one of the most significant environmental limitations to the agriculture, and it influences more than 800 million ha [1]. Because salinity stress may adversely affect crop performance [2], it is critical that researchers in molecular breeding programmes identify genes that confer salt tolerance and examine the potential for manipulating their expression in genetically modified crops. Salinity stress interferes with many physiological and metabolic processes, resulting in chlorosis, necrosis, and ion toxicity [3]. In affected plants, the short-term stress response can result in inhibition of water uptake; reduction in root growth, leaf development, and the production of new leaves; and damage to the cells of transpiring leaves [4]. Long-term stress responses can include salt sequestration in older leaves that can cause their premature senescence and a decline in enzyme activities and the photosynthesis [5, 6].

Salt stress severely interrupts the normal growth of plants and their productivity, affecting plants throughout their whole life processes [2]. Under salt stress conditions, physiological and metabolic activities are impaired by ionic and osmotic stresses, nutritional imbalances, or a combination of these factors [7, 8]. Ionic stress induces excess Na^+^ to accumulate in leaves. During periods of high salt stress, the uptake of Na^+^ competes with that of K^+^, resulting in excess sequestration of cytoplasmic Na^+^ rather than Cl^−^ within the cells [4]. Plants respond to excess Na^+^ by maintaining a high cytosolic K^+^/Na^+^ ratio and reducing their levels of cytosolic Na^+^. In the roots of plants, K^+^ absorption from soil is primarily mediated by K^+^ channels or transporters. The transcription and activities of these K^+^ channels or transporters could be induced in response to K^+^ deficiency [9]. An overabundance of Na^+^ is detected by plasma membrane sensors, which then induce an increase in the levels of cytosolic Ca^2+^ [10]. The SOS3-SOS2 protein complex activates the SOS1 protein, a plasma membrane Na^+^/H^+^ antiporter that induces the efflux of Na^+^ [11]. In addition, SOS2 regulates the activities of the V-H^+^-ATPase and NHX1 antiporters [11]. Ion homeostasis could also suppress or stimulate other related transporter activities, e.g., members of the *Arabidopsis* K^+^ transporters (AKTs) and the K^+^ (KAT)-type transporter subfamilies in *Arabidopsis thaliana* [12]. *Arabidopsis* KEA2 is the chloroplast K^+^/H^+^ antiporter that is involved in ion homeostasis [13]. All of these transporters contribute to the development of salt tolerance in plants. Osmotic imbalance introduces water deficits, reduces the expansion of the leaf area, and causes stomatal closure, which finally reduces plant growth and the rate of photosynthesis [6]. Plants achieve ion homeostasis through the salt overly sensitive (SOS) pathway [14]. Salinity stress can also drive the production of reactive oxygenic species (ROS), i.e., hydrogen peroxide (H_2_O_2_) and superoxide (O_2_.^−^) [15, 16]. Plants alleviate the effects of salt-induced oxidative stress through the upregulation of a series of enzymatic and nonenzymatic antioxidants at the subcellular level. These enzymes include peroxidase (POD), catalase (CAT), superoxide dismutase (SOD), glutathione reductase (GR), and ascorbate peroxidase (APX). The nonenzymatic antioxidants ascorbic acid (AsA), glutathione (GSH), and carotenoids also scavenger ROS [15, 17]. Additionally, it has been reported that uric acid and/or its catabolic products can eliminate ROS, thus, these compounds could be significant antioxidants in both animals and plants [18–22].

In leaf mesophyll cells, xanthine is catalyzed by xanthine dehydrogenase 1 (XDH1) to uric acid in local and systemic tissues to remove excess H_2_O_2_ from chloroplasts, therefore protecting the plants from oxidative damage [23]. Uric acid has been recognized as a potent ROS scavenger in vitro and in vivo in land animals [24–27]. In addition, certain downstream metabolites of uric acid are suggested to have antioxidative potential [19]. Uric acid infiltration into *Arabidopsis* leaves can efficiently quench the potent oxidant peroxynitrite [28]. Exogenous uric acid added to MS solid medium could prevent or significantly reduce the accumulation of chloroplast-H_2_O_2_ in the leaves of an *Arabidopsis xdh1* mutant, suggesting that uric acid functions to scavenge chloroplast-generated H_2_O_2_ [23]. Urea has been shown to be essential for the germination of *Arabidopsis* under nitrogen-limited conditions, and recent studies have also shown that uric acid [29], allantoin, and allantoate [30] can serve as the sole nitrogen source during the growth of *Arabidopsis* plants. Watanabe et al. [31] reported that pretreatment with exogenous uric acid alleviated the drought-hypersensitive phenotype of *Arabidopsis XDH*-suppressed lines, which is likely due to its ability to remove excess ROS. However, the result of exogenous xanthine in plants have not been studied, and the manner in which the concentrations of xanthine and uric acid are regulated under stress conditions also remains unknown.

The transporters responsible for the transport of xanthine and uric acid in plants are the nucleobase-ascorbate transporters (NATs). The NAT family is also known as the AzgA-like family or the nucleobase-cation symporter (NCS) family. NAT proteins are members of the NCS1 and NCS2 families in prokaryotes [32]. NAT proteins are ubiquitous among living organisms, and approximately 20 members of this family have been functionally characterized [33]. Most of them take in the nucleobase-coupled symporters of H^+^ in bacteria, fungi, and plants, or symporters of Na^+^ in suckler [34]. NATs in mammals also transport L-ascorbic acid [35]. To our knowledge, the maize leaf permease 1 (Lpe 1) and twelve *Arabidopsis* NATs (NAT1–NAT12) have been reported to transport nucleobases and uric acid substrate. Lpe 1 is thought to be a high-affinity transporter for uric acid and xanthine [36]. AtNAT1 and AtNAT12 proteins have been proven to transport adenine, guanine and uracil [37]. Eight *Arabidopsis* NATs (AtNAT1–AtNAT8) all transport xanthine [38].

Apple (*Malus* × *domestica* Borkh.) is a widely cultivated and economically important perennial fruit crop. In recent years, salinization has deleteriously affected the quality and yield of apples. Improving apple salt tolerance by molecular biological techniques has become an important method in modern breeding [39]. Furthermore, little is known about the NAT family in woody species, such as apple, or the manner in which the expression of *MdNAT* in apple is influenced by environmental stresses. We previously isolated the *NAT* gene family from the apple genome and cloned *MdNAT7*, which is significantly induced by drought and salinity stresses [40]. Here, we focused on its function in response to salinity stress. Overexpression (Oe) of *MdNAT7* increased the concentrations of xanthine and uric acid in apple, leading to enhanced tolerance to salinity stress. This phenotype can probably be attributed to an enhancement of ROS scavenging and the mediation of the ability to maintain ion homeostasis by the upregulation of antioxidant enzymes and ion transport-related gene expression in Oe plants. Our results provide evidence that overexpression of *MdNAT7* confers salt tolerance, indicting that this gene a promising candidate for future crop salt tolerance breeding. Additionally, the exogenous application of xanthine and uric acid also enhanced the resistance of apple plants to resist salinity. Xanthine and uric acid may potentially be applied in crop production to help plants resist salinity.

## Methods

### Plant materials and treatments

Transgenic and untransformed wild type (WT) tissue-cultured plants of *Malus domestica* cv. ‘Roya Gala’ (‘GL3’, Kindly provided by Prof. Zhihong Zhang at Shenyang Agricultural University, Shenyang, China) were initially grown on MS solid medium containing 0.3 mgL^− 1^ 6-benzylaminopurine (6-BA) and 0.2 mgL^− 1^ indole acetic acid (IAA) and were rooted on an MS solid medium that contained 0.5 mgL^− 1^ indole-3 butyric acid (IBA) and 0.5 mgL^− 1^ IAA. Plants were cultured under 23 °C and 60 μmolm^− 2^ s^− 1^, with a photoperiod 14 h. The apple seedlings were transferred to plastic pots that contained a mixture of soil and perlite and grown at 22 °C under a 16 h light and 8 h dark cycle after rooting on an MS solid medium. The plants were transferred to large plastic pots and kept in the glasshouse after adaptation in a growth chamber. After 3 months, plants that were healthy and uniformly sized were assigned to two experimental groups. Half of the plants were subjected to salinity using 1 L of 200 mM NaCl every 3 days [41], while the other half were treated with 1 L of water every 3 days and continued to receive standard, daily irrigation. After stress exposure for 0, 3, 6, 9, and 12 days, fully mature leaves from positions 9–12 along the stem base were sampled from three trees per treatment between 10 and 11 am, quickly frozen in liquid nitrogen and stored at − 80 °C.

To evaluate the effects of xanthine and uric acid on apple growth, similarly sized ‘GL3’ cuttings with three leaves were placed on MS solid medium for 7 days to induce root primordia. After screening concentrations of xanthine, uric acid and NaCl during a preliminary experiment, the stem tips were transferred to unadulterated MS media and MS media containing various treatments: 50 mM xanthine (Sigma-X7375, Sigma-Aldrich, St. Louis, MO, USA), 50 mM NaCl, 50 mM NaCl supplemented with 50 mM xanthine, and 50 mM NaCl combined with 20 mM uric acid (Sigma-U2625). The growth phenotype of apple plant was evaluated after 21 days of growth.

### Analysis of the signature motif and subcellular localization of MdNAT7

To characterize and analyse the signature motif of the NAT proteins, full-length protein sequences of *Aspergillus nidulans*, *Bacillus subtilis*, *Escherichia coli*, *Aspergillus nidulans*, and *Candida albicans* were downloaded from the NCBI protein database (http://www.ncbi.nlm.nih.gov/guide/).

The full coding region of MdNAT7 (without a stop codon) was cloned into the pGWB405 vector containing the green fluorescent protein (GFP) reporter gene [42]. The plasmid of 35S::MdNAT7-GFP was introduced into *Agrobacterium tumefaciens* EHA105 and then transiently transformed into tobacco (*Nicotiana benthamiana*) leaves as described by Yang et al. [43]. The expression of GFP was observed using confocal microscopy (version 2.1a; Olympus, Berlin, Germany) 2 days after agroinfiltration. The AtCBL1n:mCherry construct was used as a marker for the localization of plasma membrane proteins [44]. The primers and restriction sites used are listed in Table S1.

### Plasmids construction and apple genetic transformation

The entire coding region of *MdNAT7* was amplified with specific primers (forward primer: 5′-GCTCTAGAATGGGAGAAAATGCT-3′, XbaI site underlined; reverse primer: 5′-CCCCCGGGCTAATAATAGAAAAAT-3′, SmaI site underlined) and ligated into the pCambia121 vector using double enzyme digestion, which was driven by the maize (*Zea mays*) ubiquitin promoter and the nopaline synthase terminator. The sense and antisense sequences of the specific 200-bp coding region of *MdNAT7* were cloned into the RNAi vector (Hellsgate2) to generate the RNAi construct targeting the expression of *MdNAT7*. These constructs were transferred to *A. tumefaciens* EHA105 using electroporation [45]. *Agrobacterium*-mediated transformation was employed to create transgenic ‘Royal Gala’ apple plants [46]. Regenerated buds that were resistant to 25 mgL^− 1^ kanamycin were subcultured every 21 days. Transgenic lines were determined at DNA, RNA and protein levels. The primers and restriction sites used are listed in Table S1.

### DNA isolation, RNA extraction, qRT–PCR, and western blotting

Apple DNA was extracted using the CTAB method [47]. Total RNA was isolated from the positive lines and WT plants using the CTAB method [48] and was reverse transcribed using a Revert Aid First Strand cDNA Synthesis Kit (Thermo Scientific, Waltham, MA, USA). The quantitative real-time reverse transcriptase PCR (qRT-PCR) procedures utilized SYBR® Premix Ex Taq™ II (TliRNaseH Plus) (Takara, Dalian, China), and the assay was performed using a CFX96 TM REAL-Time System C1000 Thermal Cycler (Bio-Rad Laboratories, Foster City, CA, USA). The gene elongation factor 1α in *M. domestica* (*EF-1*α; DQ341381) was used to standardize different genes in cDNA samples [49]. The relative expression level of each gene was calculated according to the 2^-ΔΔCT^ method [50].Three biological samples were used in all experiments. The primer sequences for the analysis of gene expression are shown Table S1.

Total proteins was extracted as previously described [51]. Protein assay kits (Bio-Rad) were used to determine the concentration of proteins using bovine serum albumin as the standard. The soluble protein fraction of oxidized proteins was detected using an Oxy Blot protein oxidation detection kit (Chemicon International, Temecula, CA, USA). MdNAT7-specific monoclonal antibody against a peptide (GDARNEEFYSLPVRC) was raised in rabbits (GenScript, Nanjing, China). Clarity TM Western ECL Substrate (Bio-Rad) was used to detect the antigen-antibody complexes after incubation with a horseradish peroxidase-linked secondary antibody (CWBIO) according to the manufacturer’s instructions.

### Evaluation of stress tolerance

The electrolyte leakage (EL) of leaves was measured as described by Sun et al. [52]. The concentration of malondialdehyde (MDA) was measured as described by Heath and Packer [53]. Leaves were collected from 10 plants per treatment to analyze various physiological indexes. Chlorophyll (Chl) was extracted in 80% acetone, and chlorophyll concentrations were determined spectrophotometrically as described by Arnon [54]. The net rates of photosynthesis (Pn) were monitored using a portable photosynthesis system of LI-COR 6400 (LI-COR, Lincoln, NE, USA). The data were recorded between 9 and 10 am on sunny days by measuring the leaves at positions 9 to 12 from the base of selected plant. The Pn was evaluated from five plants per treatment. The specific experimental parameters were as described by Sun et al. [52].

### Determination of the concentrations of xanthine and uric acid in apple leaves

Xanthine and uric acid were extracted from the leaves with three biological replicates as described by Rukdee et al. [55]. Briefly, approximately 0.5 g of frozen tissue was ground to a fine powder in a mortar with liquid nitrogen and mixed with 5 mL of 10 mM ammonium hydroxide in a blender for 20 min. The supernatant was collected after centrifugation at 13,000 g for 10 min. The xanthine and uric acid in each supernatant were detected by high-performance liquid chromatography-tandem mass spectrometry (HPLC-MS/MS) on an AB SCIEX QTRAP® 5500 LC/MS/MS system [55]. Xanthine and uric acid were used as the master standards.

### Assays of antioxidant enzymes and antioxidant metabolites

Apple leaves (0.1 g) were ground separately in liquid nitrogen and then suspended in 1 mL of solution with 10 mM phosphate buffer (pH 7.0). The supernatant was collected after centrifugation (4 °C, 13,000 g, 20 min). The activities of CAT, SOD, POD, GR, and APX and the contents of AsA and GSH were measured as previously described [22, 56].

### Assessing the concentrations of sodium and potassium in leaves

After salinity treatment for 12 days, the leaf tissues of transgenic and untransformed wild-type apple plants were collected and washed three times with distilled water for measurement of the mineral concentrations. The leaf tissue was then fixed at 105 °C for 30 min, dried at 80 °C for 48 h, and ground into powder. The sodium and potassium elements were digested in solutions containing HNO_3_-HClO_4_. After the materials were filtered and then diluted with distilled water, the Na^+^ and K^+^ concentrations were analyzed using a flame photometer (M410; Sherwood Scientific, Cambridge, UK).

### Statistical analysis

All experiments were conducted in triplicate, and statistical analysis of the data from plants in the control and stress treatments was performed by one-way analysis of variance (ANOVA) using SPSS 20.0 (IBM, Inc., Armonk, NY, USA). The difference between treatments was considered statistically significant at *P* < 0.05. The data are presented as the mean plus or minus standard deviation (SD) for three replicates.

## Results

### *MdNAT7* is responsive to salt and might encode a xanthine and uric acid transporter

*MdNAT7* (MDP0000304285) encodes one of the 14 nucleobase-ascorbate transporters in *Malus*, and has a GenBank accession number of KT454524. This gene located on chromosome 8 with 1641 bp ORF and encodes 546 amino acid residues [40].

**Fig. 1:**
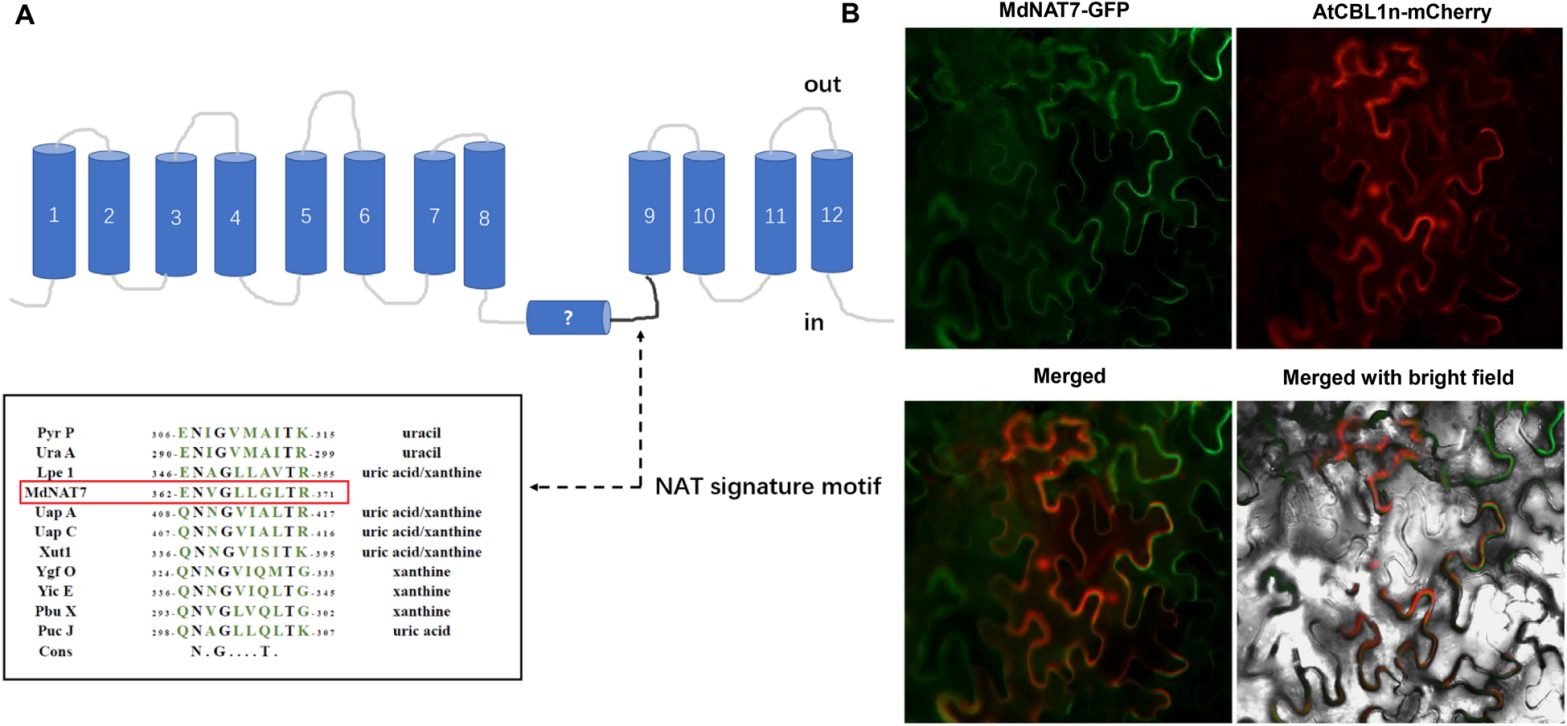
Analysis and localization of MdNAT7. **a.** Model of the transmembrane structure of NAT proteins and the NAT signature motif. Pyr P, *Bacillus subtilis* Pyr P (P31466), Ura A, *Escherichia coli* homologue Ura A (P0AGM7); Lpe 1, maize Leaf Permease 1 (NP_001150400.1); MdNAT7, *M. domestica* NAT7 (MDP0000304285); Uap A, *Aspergillus nidulans* Uap A (Q07307); Uap C, *A. nidulans* Uap C (P48777); Xut1, *Candida albicans* Xut1 (AAX22221.1); Ygf O, *E. coli* homologue YgfO (P67444); Yic E, *E. coli* homologue Yic E (P0AGM9); Pbu X, *B. subtilis* Pbu X (P42086); Puc J, *B. subtilis* Pbu J (O32139); Cons, Consensus refers to the nucleobase-ascorbate transporter motif. **b.** Localization of MdNAT7. The fusion protein of MdNAT7–GFP was transiently expressed in tobacco leaves and observed with confocal microscopy. MdNAT7–GFP fluorescence. The plasma membrane protein localization marker AtCBL1n-mCherry. Merged images. Merged with bright field. Scale bar = 50 μm

**Fig. 2:**
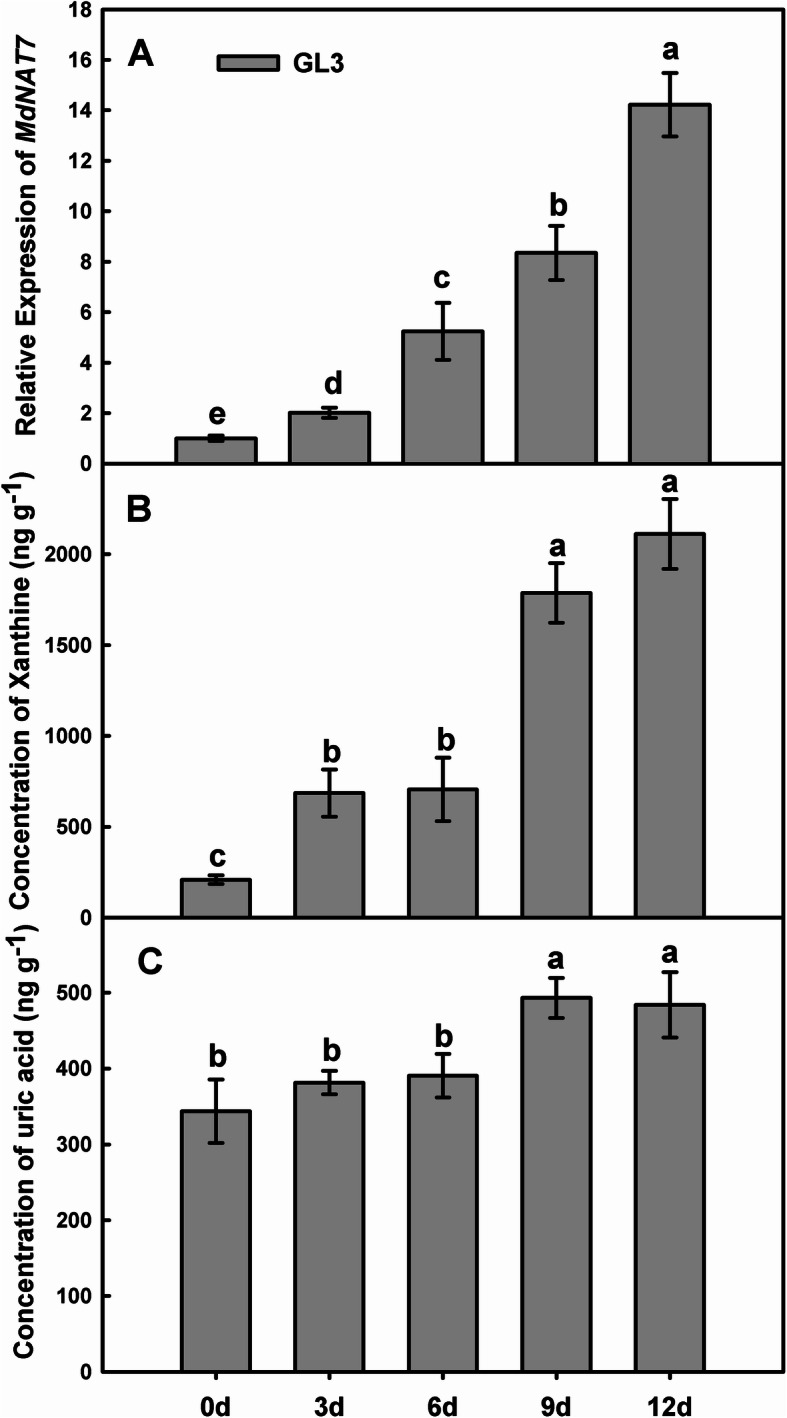
Analysis of xanthine and uric acid in apple leaves under salt stress. **a.** *MdNAT7* expression during salt stress in *Malus domestica* cv. ‘Roya Gala’ plants. **b–c.** The concentrations of xanthine and uric acid in response to salt stress in ‘Roya Gala’ plants at 0, 3, 6, 9, and 12 days. Data are the means and SDs of three replicates. Different letters indicate significant differences between treatments, according to Tukey’s multiple range tests (*P* < 0.05)

All nucleobase-ascorbate transporters contain 13–14 transmembrane segments, with a highly conserved signature motif ([Q/E/P]-N-X-G-X-X-X-X-T-[R/K/G]), which is pivotal for the NAT feature (Fig. 1a). Maize leaf permease 1 (Lpe 1) was assigned as a xanthine and uric acid high-affinity transporter [36]. MdNAT7 has a conserved signature motif (362-E-N-V-G-L-L-G-L-T-R-371) that exhibits high similarity with that of Lpe1 (Fig. 1a). A subcellular localization study indicated that the MdNAT7-GFP fusion proteins were localized to the plasma membrane (Fig. 1b). The expression of the *MdNAT7* gene was significantly upregulated upon salt stress treatment, with approximately 14-fold induction at 12 days post treatment (Fig. 2a). In addition, the potential substrates xanthine (X) and uric acid (UA) were also upregulated upon salt treatment (Fig. 2b–c).

### Exogenous xanthine and uric acid promote plant growth and alleviates salt damage in apple

**Fig. 3:**
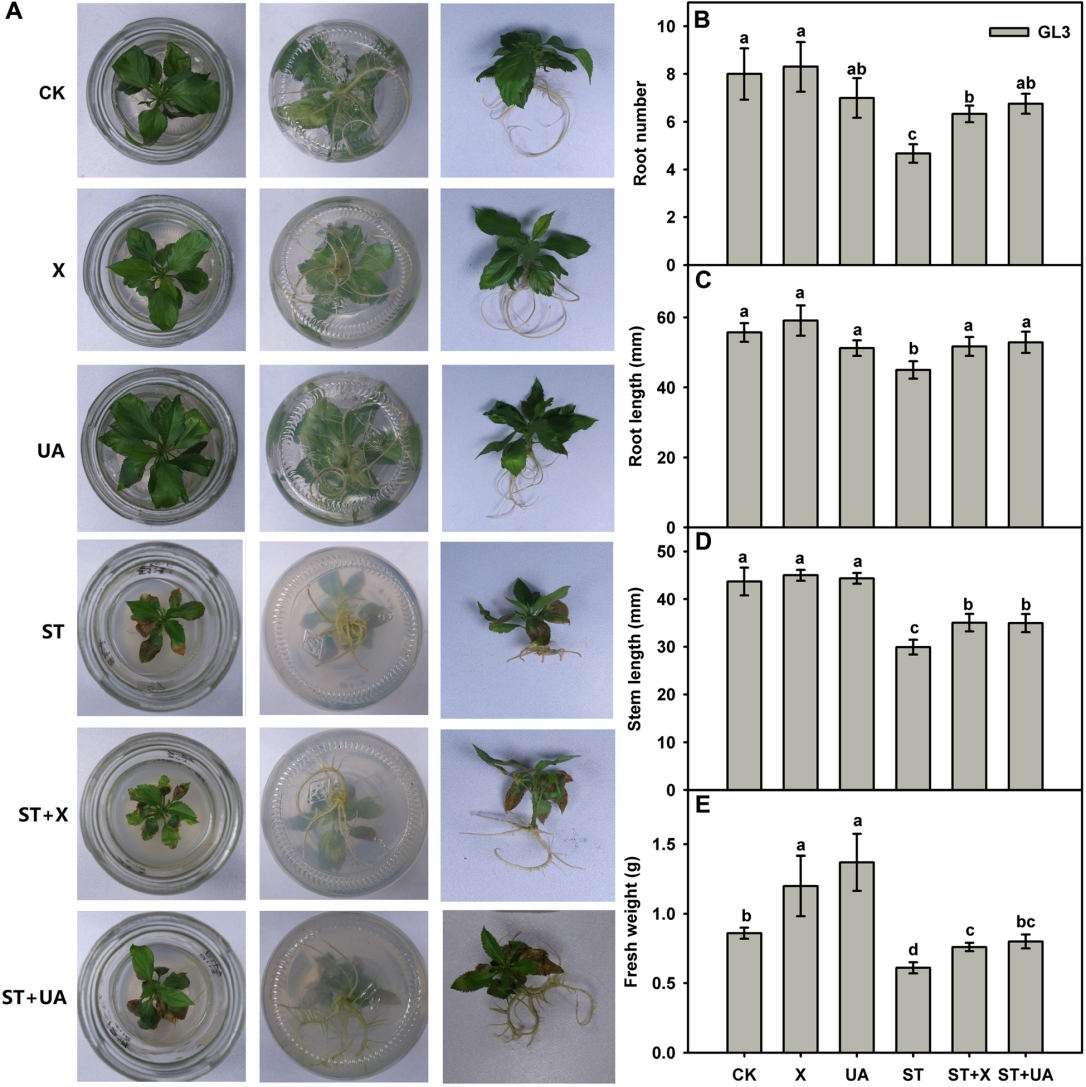
Analysis of xanthine and uric acid in apple leaves under normal and salt stress. **a.** Phenotype of tissue-cultured ‘Roya Gala’ plants in response to various treatments after 21 days of culture. **b**–**e**. Statistical analysis of tissue-cultured ‘Roya Gala’ plants in response to various treatments. The root number, root length, stem length and fresh weight of Roya Gala plants were measured at 21 days. Data are the means and SDs of three replicates. Different letters indicate significant differences between treatments, according to Tukey’s multiple range tests (P<0.05). CK, MS medium; X, MS medium with 50 mM xanthine; UA, MS medium with 50 mM uric acid. ST, MS with 50 mM NaCl; ST+X, MS with 50 mM NaCl and 50 mM xanthine; ST+UA, MS with 50 mM NaCl and 20 mM uric acid

Under normal conditions, the addition of X and UA promoted plant growth and slightly improved root elongation (Fig. 3). Exogenous uric acid added to MS solid medium could scavenge chloroplast-generated H_2_O_2_ [23]. To test whether X and UA have a role in the regulation of salt resistance, we added these compounds individually into the tissue culture media to test their functions. Plant growth and root elongation were inhibited under salt condition, while the addition of X and UA alleviated this inhibition (Fig. 3a). Under salt treatment (ST), the number of roots, root length, and fresh weight of plants decreased significantly. However, when 50 mM X was applied, the root number, root length and fresh weight increased by 1.10-, 1.09-, and 1.15-fold, respectively, compared with those under salt stress conditions. Similarly, when 20 mM UA was supplied, the number of roots, root length and fresh weight also increased by 1.35-, 1.15-, and 1.25-fold, respectively, compared with those under salt stress conditions (Fig. 3b–e).

It was noted that exogenous X and UA activated the ROS scavenging system, i.e., in ‘GL3’ apple plants, the content of H_2_O_2_ in the plants treated with ST + X and ST + UA was noticeably lower than that in the ST-treated plants, and the activities of the antioxidant enzymes SOD, POD, and CAT were much higher in the plants treated with ST + X and ST + UA than those in the plants treated with ST (Fig. S1A–D). Additionally, the expression of *MdSOS1*, *MdSOS2*, *MdSOS3*, *MdNHX1*, *MdNHX2*, *MdNHX4*, *MdNHX6*, *MdAKT1*, *MdAKT2/3, MdKAT1*, and *MdKEA2* were also higher in the plants treated with ST + X and ST + UA than in those treated with ST (Fig. S1E–O).

### Overexpression of *MdNAT7* enhances salt tolerance and concentrations of xanthine and uric acid in apple

**Fig. 4:**
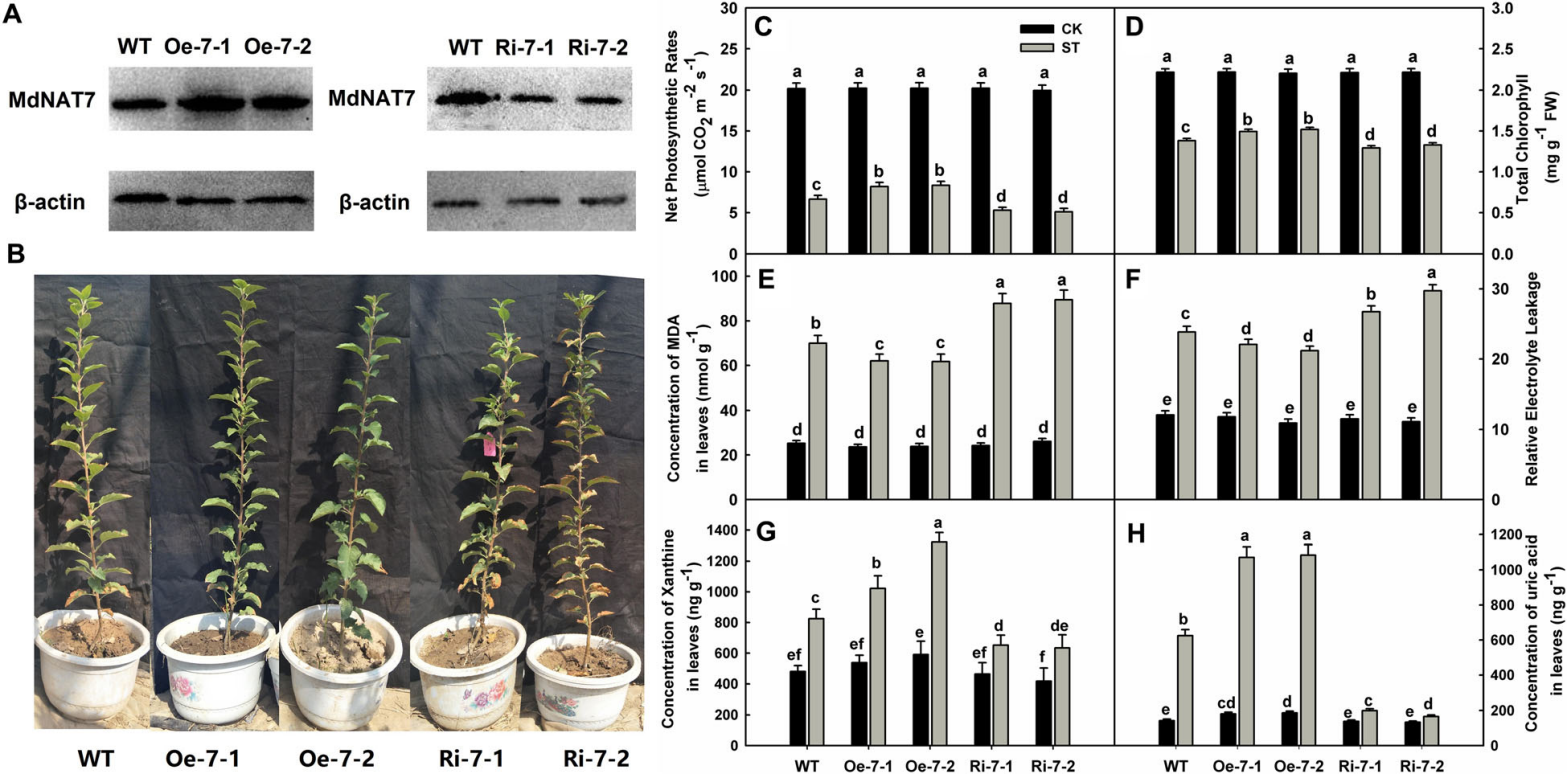
Effects of overexpression and RNAi interference of *MdNAT7* on salt tolerance in apple. **a.** Western blot assays of *MdNAT7* expression in WT, Oe and Ri plants. **b.** Phenotype of apple plants after salinity stress for 12 days. **c.** Net photosynthesis rates. **d.** Total chlorophyll. **e.** Concentration of MDA. **f.** Relative electrolyte leakage. **g**–**h**. Changes in X and UA concentrations in apple leaves under salinity stress. Three-month-old seedlings of transgenic and WT plants were irrigated with 200 mM NaCl for 12 days, and the leaves were used for physiological analyses. Different letters indicate significant differences between treatments, according to Tukey’s multiple range tests (P<0.05)

To further determine the functions of *MdNAT7*, overexpression (Oe) and RNAi (Ri) transgenic *MdNAT7* apple plants were generated. Two representative Oe lines with high *MdNAT7* expression levels (16- and 24-fold) and two Ri lines with low expression levels (0.5- and 0.2-fold) were selected for further study (Fig. 4a and S2). After exposure to salinity for 12 days, the leaves from Oe lines were less wilted and necrotic, and most remained vibrant compared with those of the WT. In contrast, the Ri lines displayed a substantial amount of wilting and necrosis (Fig. 4b). The Pn values were markedly influenced in all genotypes after treatment with 200 mM NaCl, albeit to varying degrees. For example, the rates in Oe lines were approximately 1.2-fold higher than those in the WT, while the Pn values from Ri plants were only 70% of those measured for the WT (Fig. 4c). The total concentrations of Chl in Oe-7-1, Oe-7-2, Ri-7-1, and Ri-7-2 were 1.11-, 1.22-, 0.83-, and 0.68-fold, respectively, higher than those in WT plants (Fig. 4d). The concentration of MDA was lower in Oe plants but higher in Ri lines in response to salt treatment (Fig. 4e). In addition, Oe plants showed significantly reduced EL values compared with those of the WT, while Ri plants had higher EL values than the WT (Fig. 4f). All of these data demonstrated that overexpression of *MdNAT7* caused to less physiological damage, while RNAi of *MdNAT7* led to greater physiological damage, compared with that of WT plants.

Salt stress induced the accumulation of X and UA in apple compared with that under normal conditions. Under normal conditions, the concentrations of X and UA in the leaves did not differ noticeably among genotypes (Fig. 4g–h). Compared with WT plants, the levels of X increased by 1.73- and 1.70-fold in the Oe-7-1 and Oe-7-2 lines, respectively. In contrast, these levels were reduced by 0.31- and 0.26-fold in the Ri-7-1 and Ri-7-2 lines under salt stress conditions, respectively, compared with those in the WT.

### Overexpression of *MdNAT7* leads to a lower accumulation of H_2_O_2_ under salinity stress

**Fig. 5:**
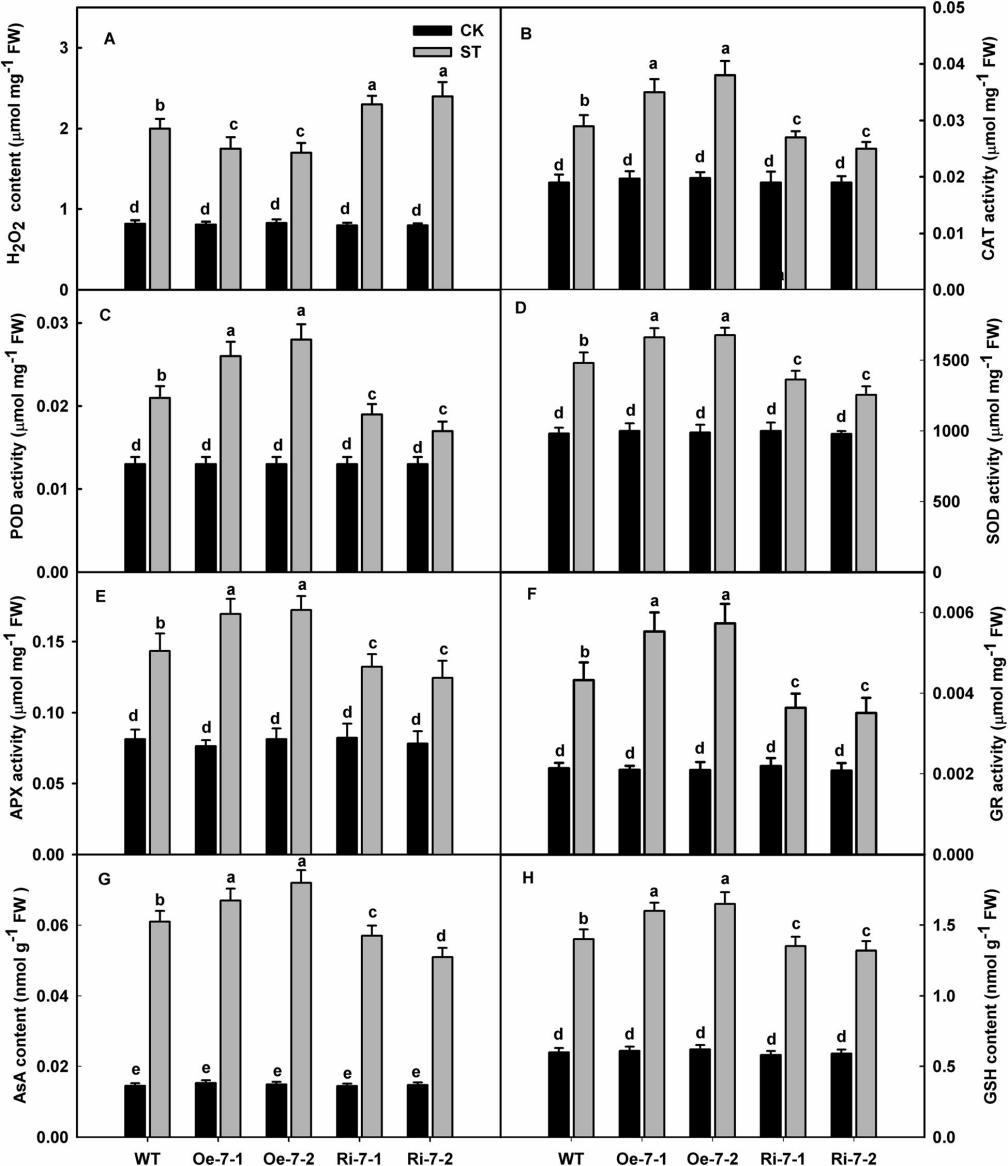
Changes in the levels of H_2_O_2_ accumulation and activities of ROS-scavenging enzymes in apple leaves under saline condition. **a**–**h**. H_2_O_2_ content, POD activity, CAT activity, SOD activity, APX activity, GR activity, AsA content, GSH content. Three-month-old seedlings of transgenic and WT plants were irrigated with 200 mM NaCl for 12 days, and the leaves were used for physiological analyses. Different letters indicate significant differences between treatments, according to Tukey’s multiple range tests (P<0.05)

Although all the genotypes tested accumulated ROS in response to salt stress, compared with WT, Oe plants had significantly less H_2_O_2_ after 12 days of treatment, while Ri plants had significantly more H_2_O_2_ (Fig. 5a). The main scavenging enzymes, POD, CAT, SOD, GR, and APX, showed obvious increases in their activities in response to the elevated accumulation of H_2_O_2_. For example, the activity of CAT increased by 1.84-, 2.00-, 1.42, and 1.31-fold for Oe-7-1, Oe-7-2, Ri-7-1, and Ri-7-2, respectively, versus the 1.53-fold increase measured in WT plants (Fig. 5b). A similar pattern was observed for the activities of POD, SOD, APX, and GR (Fig. 5c–f). These findings showed that overexpression of *MdNAT7* enhanced antioxidant activities, while RNAi reduced those activities in stressed plants. After 12 days of stress treatment, the levels of AsA and GSH were higher in Oe lines and lower in Ri lines compared with those of WT, although no difference was found between WT and transgenic plants under control conditions (Fig. 5g–h). We monitored the changes in the transcript levels of major genes in that cycle. Under salt stress conditions, the expression of *MdcAPX*, *MdDHAR1*, *MdcGR*, and *MDHAR* gradually increased, particularly in the Oe lines (Fig. S3). For example, the expression level of *MdcAPX* was 1.12- and 1.22-fold higher in Oe-7-1 and Oe-7-2, respectively, than in the WT. In contrast, the transcript levels in Ri-7-1 and Ri-7-2 were 0.52- and 0.46-fold lower than those detected in the WT.

### *MdNAT7*-overexpression lines accumulate less Na^+^ and more K^+^ than WT plants under salinity stress

**Fig. 6:**
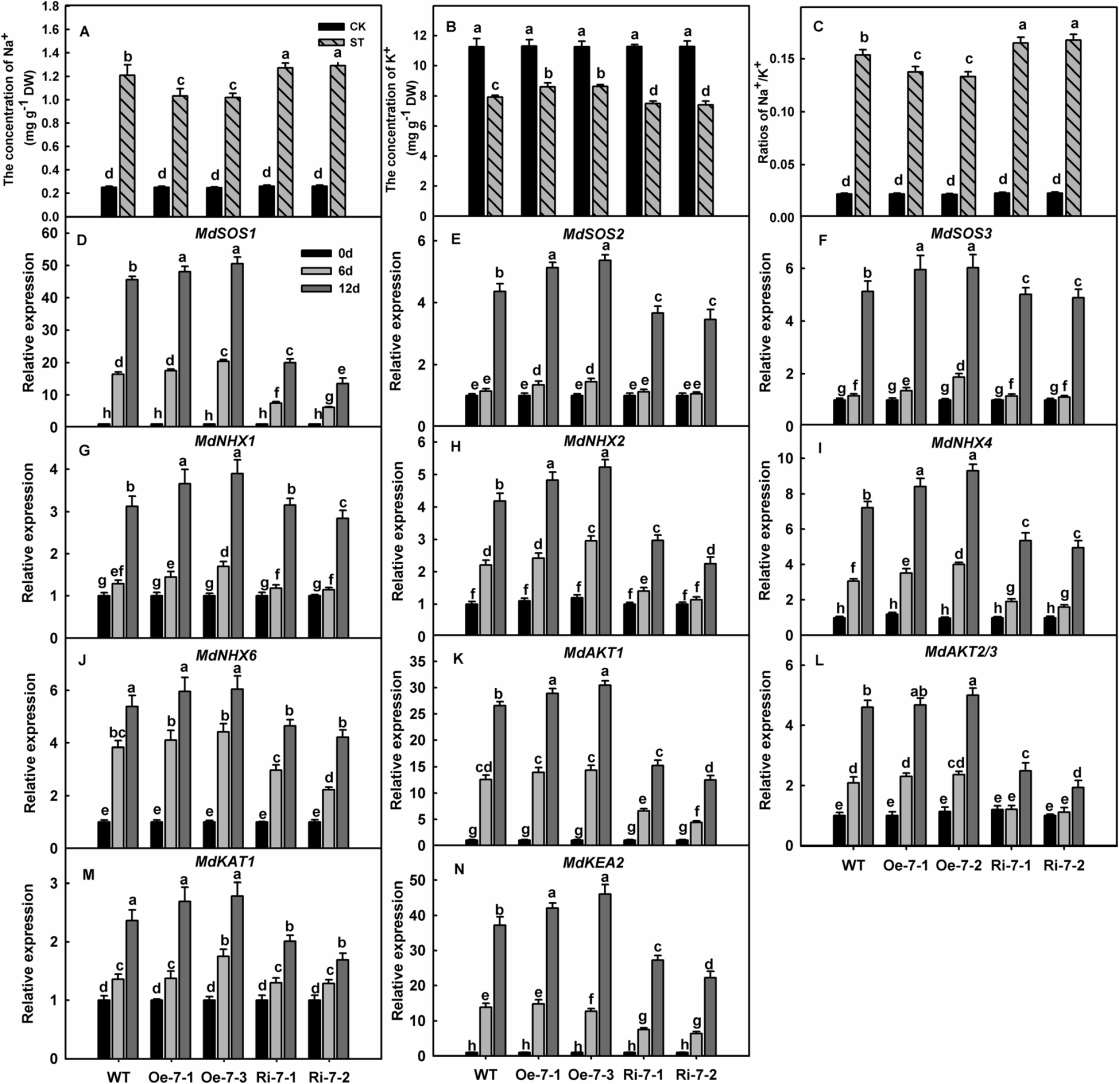
Changes in the concentrations of Na^+^ and K^+^ and salinity-related gene expression in apple leaves under salinity stress. **a.** The concentration of Na^+^. **b.** The concentration of K^+^. **c.** Ratio of Na^+^ to K^+^. **d**–**n**. The relative expression of *MdSOS1*, *MdSOS2*, *MdSOS3*, *MdNHX1*, *MdNHX2*, *MdNHX4*, *MdNHX6*, *MdAKT1*, *MdAKT2/3*, *MdKAT1*, and *MdKEA2* in apple leaves. Three-month-old seedlings of transgenic and WT plants were irrigated with 200 mM NaCl for 12 days, and the leaves were used for physiological analyses. Different letters indicate significant differences between treatments, according to and Tukey’s multiple range tests (P<0.05)

Under normal conditions, the concentrations of Na^+^ in the leaves did not differ noticeably among different genotypes. However, exposure to salt stress caused the levels of Na^+^ to be 0.86- and 0.81-fold in lines Oe-7-1 and Oe-7-2 and 1.09- and 1.11-fold in lines Ri-7-1 and Ri-7-2 compared with that in the WT, respectively (Fig. 6a). Oe plants maintained a higher concentration of K^+^ than WT plants, while the opposite was true for Ri lines (Fig. 6b). Moreover, the values for Na^+^/K^+^ were lower in Oe lines than in WT leaves under stress conditions, while those ratios were higher in the Ri lines than in the WT (Fig. 6c).

The expression of *MdSOS1*, *MdSOS2, MdSOS3, MdNHX1*, *MdNHX2*, *MdNHX4*, *MdNHX6*, *MdAKT1*, *MdAKT2/3*, *MdKAT1*, and *MdKEA2* in the leaves increased significantly in response to salinity stress. In addition, most of the genes tested were expressed at higher levels in Oe-7-1 and Oe-7-2 and at lower levels in Ri-7-1 and Ri-7-2 than in WT plants (Fig. 6d–n).

## Discussion

The nucleobase transporters and similar sequences of unknown function present in databases constitute three basic families. These are the Nucleobase Cation Symporter family 1 (NCS1), also known as the Purine-Related Transporter family (PRT), the Nucleobase-Ascorbate Transporter family (NAT or NCS2), the AzgA-like family and the so called Equilibrative Nucleoside Transporter family (ENT) [57]. The nucleobase-ascorbate transporter family is one of three known protein families, and the principal substrates for NATs are nucleobases. NATs are part of an evolutionarily widespread transport proteins family in prokaryotes, fungi, mammals, and plants [58].

We previously cloned *MdNAT7* from *M. domestica* and found that while its expression was somewhat high in young and mature apple fruits, it continued to increase during the ripening period. The transcript levels of *MdNAT7* were upregulated in response to excess NaCl and peaked after 9 days of treatment [40]. To gain new information about the mechanism by which this gene confers salt tolerance, we used transgenic apple plants to examine the effects of either overexpressing or silencing this gene. Our overall data clearly demonstrated that Oe lines exhibited improved tolerance to salt stress, while Ri plants exhibited decreased tolerance.

The NAT signature motif characteristic [Q/E/P]-N-X-G-X-X-X-X-T-[R/K/G] is a common feature of all NAT proteins, NAT proteins that tansport nucleobase or ascorbic acid could perhaps be predicted based on amino acid variations in or near the NAT signature motif. In particular, the first amino acid in the motif of known nucleobase transporters is always a Q/E, while it is a P in ascorbate transporters [59]. In our previous study, the first amino acid E is the signature motif for MdNATs [40], indicating that MdNATs are probably nucleobase transporters. *Escherichia coli* UraA knockout mutants that expressing AtNAT3 and AtNAT12 proteins were revealed to have high affinities for the transport of guanine, adenine and uracil [60]. Hunt [38] reported that all AtNATs transport xanthine. Since MdNAT7 shares the highest similarity with AtNAT3, we speculate that MdNAT7 may also transport xanthine. A highly conserved signature motif of MdNAT7 is (362-E-N-V-G-L-L-G-L-T-R-371), which exhibits a high degree of similarity with that of Lpe1 (Fig. 1a). Therefore, we hypothesize that MdNAT7 may also transport xanthine and uric acid. Fusion proteins, such as AtNAT3–GFP and AtNAT12–GFP, are located in structures that resemble the plasma membrane [37]. Similarly, MdNAT7 is also a plasma membrane protein based on our data (Fig. 1b). Similar to our previous research [40], *MdNAT7* gene expression was significantly upregulated under salt stress treatment (Fig. 2a). The potential substrates xanthine (X) and uric acid (UA) were also upregulated upon saline stress (Fig. 2b–c).

In plants, purine catabolism to xanthine is mediated by xanthine dehydrogenase (XDH), which catalyzes xanthine into uric acid, which serves as a scavenger of ROS. In leaf mesophyll cells, XDH1 catalyses xanthine to uric acid in local and systemic tissues to clear H_2_O_2_ from chloroplasts, thereby protecting plants from oxidative damage [23]. The accumulation of increased levels of xanthine as a substrate leads to higher XDH activity and more uric acid production [23]. Uric acid has long been recognized as a potent ROS scavenger in vitro and in vivo in land animals [24–27]. In *Arabidopsis*, XDH mutants, such as *Atxdh1*, exogenous uric acid or its catabolic products, can reduce natural or dark-induced early senescence [19, 42]. When *XDH*-suppressed lines were subjected to drought stress, plant growth was markedly reduced in conjunction with significantly enhanced cell death and H_2_O_2_ accumulation [31]. In the leaves of the *Arabidopsis xdh1–2* mutant, the addition of exogenous uric acid to MS solid medium completely suppressed the accumulation of H_2_O_2_ in chloroplasts [23]. Uric acid can efficiently reduce lesion formation in *Arabidopsis* leaves treated with an abiotic peroxynitrite-generating system or with a peroxynitrite solution [28]. In this study, we also discovered that exogenous xanthine and uric acid not only promoted plant growth and slightly improved the elongation of roots under normal conditions but also clearly enhanced the resistance of apple to salinity stress (Fig. 3). Exogenous X and UA activated the ROS scavenging system, upregulated several ion transporters and affected gene expression (Fig. S1). Xanthine and uric acid have potential uses in salt stress alleviation.

In this study, the overexpression lines of *MdNAT7* showed increased concentrations of xanthine and uric acid compared with those of the WT, while the Ri lines had decreased levels (Fig. 4g–h). Arabidopsis AtNAT1 and AtNAT12 have been shown to transport guanine, adenine, and uracil [37], and eight Arabidopsis NATs (AtNAT1-AtNAT8) all transport xanthine. Therefore, it is reasonable to propose that MdNAT7 functions as a xanthine and uric acid transporter in apple. The increased concentrations of xanthine and uric acid in Oe apple plants may be one reason for the improvement in their resistance to salt. Compared with WT plants, Oe lines incurred less stress-related damage, as evidenced by the lower values determined for MDA, ROS, and EL and the higher chlorophyll concentrations and photosynthesis rates (Fig. 4c–f). Furthermore, the activities of H_2_O_2_-scavenging enzymes, such as CAT, POD, SOD, APX, and GR, were augmented in the Oe lines, while the opposite was true for the Ri lines (Fig. 5).

The level of malondialdehyde, the product of lipid peroxidation, is regarded as an indicator of oxidative damage. Thus, the stability of cell membranes is extensively utilized to differentiate between salt-sensitive and salt-tolerant plants [61]. Here, the concentrations of MDA and the level of EL were prominently higher in WT than in Oe lines but were lower than those in Ri lines under salinity stress. This indicated that cell damage could be relieved by the overexpression of *MdNAT7* and aggravated when its expression was silenced. Abiotic stress leads to an increase in the production of ROS, resulting in oxidative damage to cellular components [62]. Oe apple plants accumulated less H_2_O_2_ than did WT plants after 12 days of salt stress, while oxidative damage in the Ri lines was more severe than in the WT.

Plants use enzymatic and nonenzymatic antioxidants to protect cells and subcellular systems from ROS damage [63]. We showed here that the activities of POD and CAT were obviously higher in the Oe lines than in the WT under saline condition. APX, as the most important scavenger of H_2_O_2_ in green tissues, functions in the AsA–GSH cycle and is located in diverse cellular compartments [64]. Overexpression of *MdNAT7* upregulated the activities of APX and GR and the expression of *MdcAPX*, *MdMDHAR*, *MdDHAR1*, and *MdcGR* in the AsA-GSH cycle system (Fig. S3). This could explain why Oe lines contained more AsA and GSH than the WT, while Ri lines had the least amount when subjected to salt stress. Under normal conditions, the expression of those genes showed no difference between transgenic and WT plants, similar to the contents of AsA and GSH and the activity of antioxidant enzymes. This trend in the concentrations of AsA and GSH and interconversions between their oxidized and reduced forms is also consistent with the changes in transcriptional pattern. The total levels of AsA and total GSH were prominently improved in Oe plants under saline condition. After 12 days of salt stress, the levels of AsA were higher in the Oe lines than in the WT, probably because the Oe lines had a more activated antioxidant system (Fig. 5g–h). Salinity stress affects photosynthetic processes when salt accumulates in the tip [1], and the concentrations of chlorophyll and carotenoids decrease, even in halophyte plants [65–67]. The rate of photosynthesis can also decline owing to the production of ROS, and repairs to Photosystem II are prevented owing to an imbalance in the chloroplast redox system [68]. The negative influence of photosynthesis intracellular ROS can be alleviated by engineering plants to increase the production of ROS-scavenging enzymes, such as, APX and CAT, by improving the levels of GSH and AsA antioxidants [68]. The strengthening of Pn that we observed in Oe plants could have resulted from their more robust antioxidant system.

MdNAT7 regulated not only the genes involved in ROS scavenging but also the genes involved in ion transport. Plant responses to saline conditions are modulated by many different genes that help confer tolerance [69]. We detected the transcript levels of genes that encoded such transporters – MdSOS1, MdSOS2, MdSOS3, MdNHX1, MdNHX2, MdNHX4, MdNHX6, MdAKT1, MdAKT2/3, MdKAT1, and MdKEA2 – and found that they were induced to remarkable levels in all genotypes under saline conditions. Compared with WT plants, overexpression of *MdNAT7* also showed higher transcript levels for genes related to salt tolerance, while the opposite was noted for the Ri lines (Fig. 6d–n). The same pattern was noted for the concentrations of Na^+^ and K^+^, with levels of the former found at lower levels in Oe plants and amounts of the latter found at higher levels under saline conditions (Fig. 6a–c). Therefore, overexpression of *MdNAT7* reduced Na^+^/K^+^ values in the leaves, while interference with *MdNAT7* enhanced them.

## Conclusion

In summary, apple MdNAT7 might transport xanthine and uric acid that could scavenge ROS more efficiently by enhancing H_2_O_2_-scavenging enzymes and the concentrations of xanthine and uric acid under salt stress. Additionally, MdNAT7 could also maintain ion homeostasis by activating the expression of genes encoding ion transporters using certain unknown mechanisms. Our results provide evidence that overexpression of *MdNAT7* confers salt tolerance, rendering this gene a promising candidate for future crop salt tolerance breeding in apple rootstocks, although general attitudes towards genetic engineering are prudent. Meanwhile, xanthine and uric acid have the potential to be applied in crop production to resist salinity stress.

## Supplementary Information

**Additional file 1: Table S1**. Primers used in this study.

**Additional file 2: Fig. S1**. Analysis of H_2_O_2_ levels, ROS-scavenging enzyme activities, and the expression of salt-related genes in ‘Roya Gala’ plants. **A**–**D**. H_2_O_2_ content, CAT activity, POD activity, SOD activity in apple leaves. **E**–**O**. Transcript levels of salt-related transporter genes *MdSOS1*, *MdSOS2*, *MdSOS3*, *MdNHX1*, *MdNHX2*, *MdNHX4*, *MdNHX6*, *MdAKT1*, *MdAKT2/3*, *MdKAT1*, and *MdKEA2* in tissue-cultured ‘Roya Gala’ plant leaves after 21 days of different stress treatments. Data are the means and SDs of three replicates. Different letters indicate significant differences between treatments according to Tukey’s multiple range tests (P<0.05). ST, MS with 50 mM NaCl; ST+X, MS with 50 mM NaCl and 50 mM xanthine; ST+UA, MS with 50 mM NaCl and 20 mM uric acid.

**Additional file 3: Fig. S2**. PCR, qRT-PCR and western blot analysis of the transgenic apple plants. **A.** PCR results for Ri and Oe transgenic apple plants detection. M, molecular marker DL2000; V1, positive vector that contains the Hellsgate2-MdNAT7 plasmid; Ri-7-1 and Ri-7-2, *MdNAT7*-Ri transgenic lines; WT, wild type; V2, positive vector that contains the pCambia121-MdNAT7 plasmid; Oe-7-1 and Oe-7-2, *MdNAT7*-Oe transgenic lines. **B.** *MdNAT7* expression in Oe and Ri plants by qRT-PCR. **C.** Western blot analysis of MdNAT7 protein in WT and Oe transgenic apple plants. **D.** Western blot analysis of β-actin protein in WT and Oe transgenic apple plants. **E.** Western blot analysis of MdNAT7 protein in WT and Ri transgenic apple plants. **F.** Western blot analysis of β-actin protein in WT and Ri transgenic apple plants. Apple leaf samples from WT and transgenic apple plants were collected under normal growth conditions. Data are the means and SDs of three replicates. Asterisks indicate significant differences between WT and transgenic lines according to Tukey’s multiple range tests (*P* < 0.05).

**Additional file 4: Fig. S3.** Changes in the levels of transcripts for the genes involved in the AsA–GSH cycle during the salt stress period: **A**–**D.** *MdcAPX*, *MdDHAR1*, *MdcGR*, and *MdMDHAR* in plant leaves. Measurements were made at 0, 6 and 12 days of treatment. Data are the means and SDs of three replicates. Different letters indicate significant differences between treatments according to Tukey’s multiple range tests (*P* < 0.05).

## Data Availability

All data generated or analysed during this study are included in this published article and its supplementary information files. The datasets used and/or analysed during the current study are available from the corresponding author on reasonable request.

## Abbreviations

XDH: Xanthine dehydrogenase
NAT: Nucleobase-ascorbate transporter
NCS: Nucleobase-cation symporter
SOS: Salt overly sensitive
NHX: Na^+^/H^+^ reverse transporter
AKT: Arabidopsis K^+^ transporter
KAT: K^+^ transporters
KEA: Chloroplast K^+^/H^+^ antiporter
ROS: Reactive oxygen species
H_2_O_2_: Hydrogen peroxide
O_2_: Superoxide
POD: Peroxidase
CAT: Catalase
SOD: Superoxide dismutase
GR: Glutathione reductase
APX: Ascorbate peroxidase
AsA: Ascorbic acid
GSH: Glutathione
EL: Electrolyte leakage
MDA: Malondialdehyde
Chl: Chlorophyll
Pn: The net rates of photosynthesis
Lpe 1: Maize leaf permease 1
WT: wild type
ANOVA: analysis of variance
SD: Standard deviation
HPLC-MS/MS: High performance liquid chromatography-tandem mass spectrometry
qRT-PCR: Quantitative real-time reverse transcriptase PCR
IAA: Indole acetic acid
IBA: Indole-3-butyric acid
6-BA: 6-benzylaminopurine.

## References

[1] Munns R, Tester M (2008). Mechanisms of salinity tolerance. Annu Rev Plant Biol.

[2] Grattan SR, Grieve CM (1998). Salinity-mineral nutrient relations in horticultural crops. Sci Hortic-Amsterdam.

[3] Testerink C, Munnik T (2011). Molecular, cellular, and physiological responses to phosphatidic acid formation in plants. J Exp Bot.

[4] Muchate NS, Nikalje GC, Rajurkar NS, Suprasanna P, Nikam TD (2016). Plant Salt Stress: Adaptive Responses, Tolerance Mechanism and Bioengineering for Salt Tolerance. Botanical Rev.

[5] Munns R (2005). Genes and salt tolerance: bringing them together. New Phytol.

[6] Roy SJ, Negrao S, Tester M (2014). Salt resistant crop plants. Curr Opin Biotechnol.

[7] Ashraf M (2004). Some important physiological selection criteria for salt tolerance in plants. Flora.

[8] Slama I, Abdelly C, Bouchereau A, Flowers T, Savoure A (2015). Diversity, distribution and roles of osmoprotective compounds accumulated in halophytes under abiotic stress. Ann Bot-London.

[9] Wang Y, Wu WH (2013). Potassium Transport and Signaling in Higher Plants. Annu Rev Plant Biol.

[10] Mahajan S, Pandey GK, Tuteja N (2008). Calcium- and salt-stress signaling in plants: shedding light on SOS pathway. Arch Biochem Biophys.

[11] Zhu JK (2003). Regulation of ion homeostasis under salt stress. Curr Opin Plant Biol.

[12] Ardie SW, Liu S, Takano T (2010). Expression of the AKT1-type K(+) channel gene from Puccinellia tenuiflora, PutAKT1, enhances salt tolerance in Arabidopsis. Plant Cell Rep.

[13] Aranda-Sicilia MN, Cagnac O, Chanroj S, Sze H, Rodriguez-Rosales MP, Venema K (2012). Arabidopsis KEA2, a homolog of bacterial KefC, encodes a K(+)/H(+) antiporter with a chloroplast transit peptide. Biochim Biophys Acta.

[14] Zhu JK (2002). Salt and drought stress signal transduction in plants. Annu Rev Plant Biol.

[15] Jithesh MN, Prashanth SR, Sivaprakash KR, Parida AK (2006). Antioxidative response mechanisms in halophytes: their role in stress defence. J Genet.

[16] Luo MB, Liu F (2011). Salinity-induced oxidative stress and regulation of antioxidant defense system in the marine macroalga Ulva prolifera. J Exp Marine Biol Ecol.

[17] Gill SS, Tuteja N (2010). Reactive oxygen species and antioxidant machinery in abiotic stress tolerance in crop plants. Plant Physiol Biochem.

[18] Becker BF, Reinholz N, Ozcelik T, Leipert B, Gerlach E (1989). Uric acid as radical scavenger and antioxidant in the heart. Pflugers Arch.

[19] Brychkova G, Alikulov Z, Fluhr R, Sagi M (2008). A critical role for ureides in dark and senescence-induced purine remobilization is unmasked in the Atxdh1 Arabidopsis mutant. Plant J.

[20] Kim YS, Nam HJ, Chung HY, Kim ND, Ryu JH, Lee WJ, Arking R, Yoo MA (2001). Role of xanthine dehydrogenase and aging on the innate immune response of Drosophila. J Am Aging Assoc.

[21] Valko M, Leibfritz D, Moncol J, Cronin MTD, Mazur M, Telser J (2007). Free radicals and antioxidants in normal physiological functions and human disease. Int J Biochem Cell B.

[22] Wang P, Yin LH, Liang D, Li C, Ma FW, Yue ZY (2012). Delayed senescence of apple leaves by exogenous melatonin treatment: toward regulating the ascorbate-glutathione cycle. J Pineal Res.

[23] Ma X, Wang W, Bittner F, Schmidt N, Berkey R, Zhang L, King H, Zhang Y, Feng J, Wen Y (2016). Dual and Opposing Roles of Xanthine Dehydrogenase in Defense-Associated Reactive Oxygen Species Metabolism in Arabidopsis. Plant Cell.

[24] Ames BN, Cathcart R, Schwiers E, Hochstein P (1981). Uric acid provides an antioxidant defense in humans against oxidant- and radical-caused aging and cancer: a hypothesis. Proc Natl Acad Sci.

[25] Kaur H, Halliwell B (1990). Action of biologically-relevant oxidizing species upon uric acid. Identification of uric acid oxidation products. Chem Biol Interact.

[26] Becker BF (1993). Towards the physiological function of uric acid. Free Radic Biol Med.

[27] Hilliker AJ, Duyf B, Evans D, Phillips JP (1992). Urate-null rosy mutants of Drosophila melanogaster are hypersensitive to oxygen stress. Proc Natl Acad Sci U S A.

[28] Alamillo JM, Garcia-Olmedo F (2001). Effects of urate, a natural inhibitor of peroxynitrite-mediated toxicity, in the response of Arabidopsis thaliana to the bacterial pathogen Pseudomonas syringae. Plant J.

[29] Nakagawa A, Sakamoto S, Takahashi M, Morikawa H, Sakamoto A (2007). The RNAi-mediated silencing of xanthine dehydrogenase impairs growth and fertility and accelerates leaf senescence in transgenic Arabidopsis plants. Plant Cell Physiol.

[30] Todd CD, Polacco JC (2006). AtAAH encodes a protein with allantoate amidohydrolase activity from Arabidopsis thaliana. Planta.

[31] Watanabe S, Nakagawa A, Izumi S, Shimada H, Sakamoto A (2010). RNA interference-mediated suppression of xanthine dehydrogenase reveals the role of purine metabolism in drought tolerance in Arabidopsis. FEBS Lett.

[32] Saier MH, Yen MR, Noto K, Tamang DG, Elkan C (2009). The Transporter Classification Database: recent advances. Nucleic Acids Res.

[33] Frillingos S (2012). Insights to the evolution of Nucleobase-Ascorbate Transporters (NAT/NCS2 family) from the Cys-scanning analysis of xanthine permease XanQ. Int J Biochem Mol Biol.

[34] Kosti V, Lambrinidis G, Myrianthopoulos V, Diallinas G, Mikros E (2012). Identification of the substrate recognition and transport pathway in a eukaryotic member of the nucleobase-ascorbate transporter (NAT) family. PLoS One.

[35] Gournas C, Papageorgiou I, Diallinas G (2008). The nucleobase-ascorbate transporter (NAT) family: genomics, evolution, structure-function relationships and physiological role. Mol Biosyst.

[36] Argyrou E, Sophianopoulou V, Schultes N, Diallinas G (2001). Functional characterization of a maize purine transporter by expression in Aspergillus nidulans. Plant Cell.

[37] Niopek-Witz S, Deppe J, Lemieux MJ, Mohlmann T (2014). Biochemical characterization and structure-function relationship of two plant NCS2 proteins, the nucleobase transporters NAT3 and NAT12 from Arabidopsis thaliana. Biochim Biophys Acta.

[38] Hunt KA. Functional characterization of the nucleobase-ascorbate transporter family of Arabidopsis thaliana. Purdue University. 2013:239–50.

[39] Feng Y, Liu J, Zhai L, Gan Z, Zhang G, Yang S, Wang Y, Wu T, Zhang X, Xu X. Natural variation in cytokinin maintenance improves salt tolerance in apple rootstocks. Plant Cell Environ. 2019;42(2). 10.1111/pce.13403 29989184

[40] Sun TT, Jia DF, Huang LL, Shao Y, Ma FW (2016). Comprehensive genomic identification and expression analysis of the nucleobase-ascorbate transporter (NAT) gene family in apple. Sci Hortic-Amsterdam.

[41] Li C, Wei Z, Liang D, Zhou S, Li Y, Liu C, Ma F (2013). Enhanced salt resistance in apple plants overexpressing a Malus vacuolar Na+/H+ antiporter gene is associated with differences in stomatal behavior and photosynthesis. Plant Physiol Biochem.

[42] Nakagawa T, Ishiguro S, Kimura T (2009). Gateway vectors for plant transformation. Plant Biotechnol.

[43] Yang Y, Li R, Qi M (2000). In vivo analysis of plant promoters and transcription factors by agroinfiltration of tobacco leaves. Plant J.

[44] Batistic O, Waadt R, Steinhorst L, Held K, Kudla J (2010). CBL-mediated targeting of CIPKs facilitates the decoding of calcium signals emanating from distinct cellular stores. Plant J.

[45] Hood EE, Jen G, Kayes L, Kramer J, Fraley RT, Chilton MD (1984). Restriction Endonuclease Map of pTi Bo542, a Potential Ti Plasmid Vector for Genetic Engineering of Plants. Nat Biotechnol.

[46] Dai HY, Li WR, Han GF, Yang Y, Ma Y, Li H, Zhang ZH (2013). Development of a seedling clone with high regeneration capacity and susceptibility to Agrobacterium in apple. Sci Hortic-Amsterdam.

[47] Modgil G, Havas T, Mellis C (2005). Idiopathic subglottic stenosis and the relationship to menses in a 12-year-old girl. J Paediatr Child Health.

[48] Chang S, Puryear J, Cairney J (1993). A simple and efficient method for isolating RNA from pine trees. Plant Mol Biol Rep.

[49] Bowen J, Ireland HS, Crowhurst R, Luo Z, Watson AE, Foster T, Gapper N, Giovanonni JJ, Mattheis JP, Watkins C (2014). Selection of low-variance expressed Malus x domestica (apple) genes for use as quantitative PCR reference genes (housekeepers). Tree Genet Genomes.

[50] Livak KJ, Schmittgen TD (2001). Analysis of relative gene expression data using real-time quantitative PCR and the 2(−Delta Delta C(T)) Method. Methods.

[51] Zhou J, Wang J, Cheng Y, Chi YJ, Fan B, Yu JQ, Chen Z (2013). NBR1-mediated selective autophagy targets insoluble ubiquitinated protein aggregates in plant stress responses. PLoS Genet.

[52] Sun TT, Pei TT, Zhang ZJ, Li MJ, Huang LL, Li CY, Shi XY, Zhan MH, Cao XY, Ma FW (2018). Activated Expression of PHT Genes Contributes to Osmotic Stress Resistance under Low Phosphorus Levels in Malus. J Am Soc Hortic Sci.

[53] Heath RL, Packer L (1968). Photoperoxidation in isolated chloroplasts. I. Kinetics and stoichiometry of fatty acid peroxidation. Arch Biochem Biophys.

[54] Arnon DI (1949). Copper Enzymes in Isolated Chloroplasts. Polyphenoloxidase in Beta vulgaris. Plant Physiol.

[55] Rukdee N, Rojsanga P, Phechkrajang CM (2015). Development and Validation of LC-MS/MS Method for Quantitative Determination of Adenosine, Guanosine, Xanthine and Uric acid in Widely Consumed Vegetables in Thailand. Nat Prod Commun.

[56] Zhang J, Niu J, Duan Y, Zhang M, Liu J, Li P, Ma F (2015). Photoprotection mechanism in the ‘Fuji’ apple peel at different levels of photooxidative sunburn. Physiol Plant.

[57] Diallinas G, Gournas C (2008). Structure-function relationships in the nucleobase-ascorbate transporter (NAT) family: lessons from model microbial genetic systems. Channels (Austin).

[58] de Koning H, Diallinas G (2000). Nucleobase transporters (review). Mol Membr Biol.

[59] Maurino VG, Grube E, Zielinski J, Schild A, Fischer K, Flugge UI (2006). Identification and expression analysis of twelve members of the nucleobase-ascorbate transporter (NAT) gene family in Arabidopsis thaliana. Plant Cell Physiol.

[60] Menaldo DL, Bernardes CP, Santos-Filho NA, Moura Lde A, Fuly AL, Arantes EC, Sampaio SV (2012). Biochemical characterization and comparative analysis of two distinct serine proteases from Bothrops pirajai snake venom. Biochimie.

[61] Hernandez JA, Almansa MS (2002). Short-term effects of salt stress on antioxidant systems and leaf water relations of pea leaves. Physiol Plant.

[62] Kasukabe Y, He LX, Nada K, Misawa S, Ihara I, Tachibana S (2004). Overexpression of spermidine synthase enhances tolerance to multiple environmental stresses and up-regulates the expression of various stress regulated genes in transgenic Arabidopsis thaliana. Plant Cell Physiol.

[63] St Clair SB, Lynch JP (2004). Photosynthetic and antioxidant enzyme responses of sugar maple and red maple seedlings to excess manganese in contrasting light environments. Funct Plant Biol.

[64] Shigeoka S, Ishikawa T, Tamoi M, Miyagawa Y, Takeda T, Yabuta Y, Yoshimura K (2002). Regulation and function of ascorbate peroxidase isoenzymes. J Exp Bot.

[65] Duarte B, Santos D, Marques JC, Cacador I (2013). Ecophysiological adaptations of two halophytes to salt stress: Photosynthesis, PS II photochemistry and anti-oxidant feedback - Implications for resilience in climate change. Plant Physiol Bioch.

[66] Stepien P, Johnson GN (2009). Contrasting responses of photosynthesis to salt stress in the glycophyte Arabidopsis and the halophyte thellungiella: role of the plastid terminal oxidase as an alternative electron sink. Plant Physiol.

[67] Parida A, Das AB, Das P (2002). NaCl stress causes changes in photosynthetic pigments, proteins, and other metabolic components in the leaves of a true mangrove, Bruguiera parviflora, in hydroponic cultures. J Plant Biol.

[68] Gururani MA, Venkatesh J, Tran LS (2015). Regulation of Photosynthesis during Abiotic Stress-Induced Photoinhibition. Mol Plant.

[69] Zhang B, Liu K, Zheng Y, Wang Y, Wang J, Liao H (2013). Disruption of AtWNK8 enhances tolerance of Arabidopsis to salt and osmotic stresses via modulating proline content and activities of catalase and peroxidase. Int J Mol Sci.

