# Supplementary material for: Exogenous application of xanthine and uric acid and nucleobase-ascorbate transporter MdNAT7 expression regulate salinity tolerance in apple
Source: BMC Plant Biol. 2021 Jan 19;21:52. doi: 10.1186/s12870-021-02831-y (PMC7816448; doi:10.1186/s12870-021-02831-y)
Supplement: Supplementary file 1 — Additional file 1: Table S1. Primers used in this study. [file 12870_2021_2831_MOESM1_ESM.docx]

Table S1 Primers used in this study

| Name | Sequence (5’-3’) | Purpose |
| --- | --- | --- |
| OeNAT7 | F: GCTCTAGAATGGGAGAAAATGCT | Vector construction for overexpression plant transformation |
|  | R: CCCCCGGGCTAATAATAGAAAAAT |  |
| Test primer | F: GCTCTAGAATGGGAGAAAATGC | Test for overexpression plant transformation |
|  | R:CCGGGTGGTCAGTCCCT |  |
| RiNAT7 | F: AAAGCAGGCTCCAGGTTTGC | Vector construction for RNAi-mediated silence plant transformation |
|  | R: AGAAAGCTGGGTTCACAGTTG |  |
| NAT7-405 | F: AAAGCAGGCTCCATGGGAGAAAATGCT | Vector construction for subcellular localization of *MdNAT7* gene |
|  | R: AGAAAGCTGGGTTCTAATAATAGAAAAAT |  |
| attb1 | F: GGGGACAAGTTTGTACAAAAAAGCAGGCTNN | Universal primer |
| attb2 | R: GGGGACCACTTTGTACAAGAAAGCTGGGTN |  |
| MdNAT7-PYES2-BamHI-F | GCTTGGTACCGAGCTCGGATCCATGGGAGAAAATGCTC | Vector construction for pYES2.0-MdNAT7 |
| MdNAT7-PYES2-Xba I-R | TACATGATGCGGCCCTCTAGACTAATAATAGAAAAATC |  |
| qMdNAT7 | F: CTCTGCATTGGGCTTGTATGGTC | Quantitative expression of *MdNAT7* |
|  | R: AGACATGGCTCGCTCGGAATAT |  |
| qMdSOS1 | F: TCCGGTTAATCCATCACACACCGT | Quantitative expression of *MdSOS1* |
|  | R: TTTGCTGCCCTGGAGGATTTGTTG |  |
| qMdSOS2 | F:CAAAAGCACCATTCTCAAGCAC | Quantitative expression of *MdSOS2* |
|  | R:CCGACCAGCCAAAACCTCT |  |
| qMdSOS3 | F:AAGGCAAGGCGGCAGTTT | Quantitative expression of *MdSOS3* |
|  | R:GCGAGGCATTGGGATGAA |  |
| qMdNHX1 | F:AAGCGACAGTCCTGGAACATCAGT | Quantitative expression of *MdNHX1* |
|  | R:TATTATCACTTGCTGCCGGAGGCT |  |
| qMdNHX2 | F: ATGCGTGGCTCTGTTTCAAT | Quantitative expression of *MdNHX2* |
|  | R: AACTGTGATGGTGCTGGTGA |  |
| qMdNHX4 | F: ATCACCAAAACCACCAACCA | Quantitative expression of *MdNHX4* |
|  | R: GCCACACTTCTTAGGCAACG |  |
| qMdNHX6 | F: AGCACAGCGTCATTCACAG | Quantitative expression of *MdNHX6* |
|  | R: ATGGAAACCCCCTCTTGTAG |  |
| qMdAKT1 | F: GCGGAGACGAAAAGTCCTAA | Quantitative expression of *MdAKT1* |
|  | R: AGTGGGAGCAGCACAAGTTT |  |
| qMdAKT2/3 | F: TTCAAGGGAAACACTTCTGC | Quantitative expression of *MdAKT2/3* |
|  | R: TCTCTCTCCATCTCACAATCAA |  |
| qMdKAT1 | F: ATGGGCAAGATCAAGTAAGTCACA | Quantitative expression of *MdKEA1* |
|  | R: GTCAGAGCCAATCGACCAAGTAT |  |
| qMdKEA2 | F: GCTGTCAATCAGGGAATAATGA | Quantitative expression of *MdKEA2* |
|  | R: CACCTCAAAACGAGAAGCAA |  |
| qMdcAPX | F: AACTACAAGGGATGAAGCC | Quantitative expression of *MdcAPX* |
|  | R: CAACGAGGATGATAACCAG |  |
| qMdMDHAR | F: CCATACTTCTATTCCCGCTCCT | Quantitative expression of *MdMDHAR* |
|  | R: CGACCACCTTCCCGTCTTT |  |
| qMdDHAR1 | F: AGTGGACGGTTCCAGCAGA | Quantitative expression of *MdDHAR1* |
|  | R: TTCCCATCCCGCAATCAC |  |
| qMdcGR | F: GTTCAGCGACAAGGCGTAT | Quantitative expression of *MdcGR* |
|  | R: TCAACCGATTTCCATTTCC |  |
| *EF-1α* | F: ATTCAAGTATGCCTGGGTGC | Real-time PCR using *Malus* *EF-1α* as reference gene |
|  | R: CAGTCAGCCTGTGATGTTCC |  |
