# Supplementary figures and images for: Exogenous application of xanthine and uric acid and nucleobase-ascorbate transporter MdNAT7 expression regulate salinity tolerance in apple
Source: BMC Plant Biol. 2021 Jan 19;21:52. doi: 10.1186/s12870-021-02831-y (PMC7816448; doi:10.1186/s12870-021-02831-y)

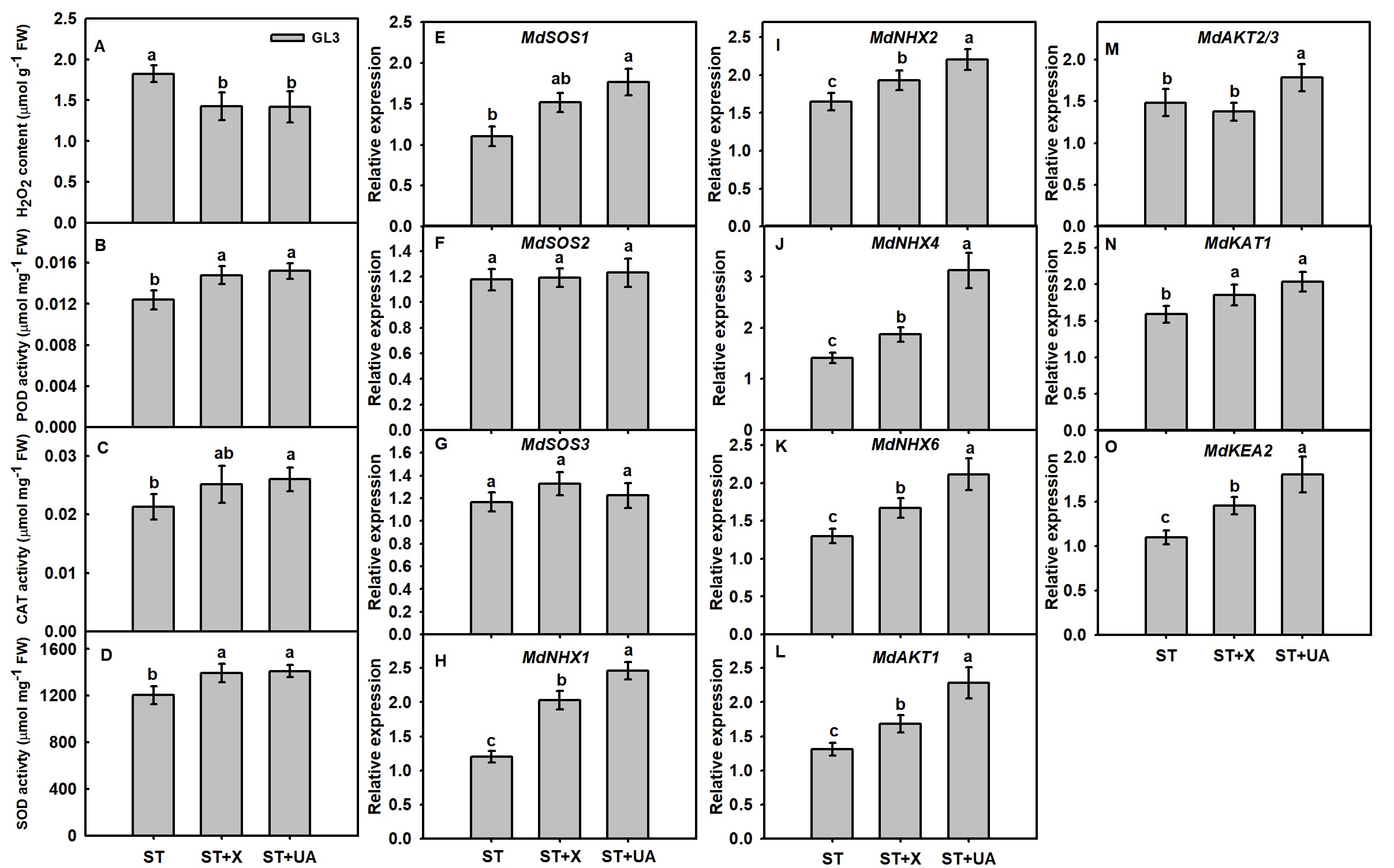

Supplement: Supplementary file 2 — Additional file 2: Fig. S1. Analysis of H2O2 levels, ROS-scavenging enzyme activities, and the expression of salt-related genes in ‘Roya Gala’ plants. A–D. H2O2 content, CAT activity, POD activity, SOD activity in apple leaves. E–O. Transcript levels of salt-related transporter genes MdSOS1, MdSOS2, MdSOS3, MdNHX1, MdNHX2, MdNHX4, MdNHX6, MdAKT1, MdAKT2/3, MdKAT1, and MdKEA2 in tissue-cultured ‘Roya Gala’ plant leaves after 21 days of different stress treatments. Data are the means and SDs of three replicates. Different letters indicate significant differences between treatments according to Tukey’s multiple range tests (P<0.05). ST, MS with 50 mM NaCl; ST+X, MS with 50 mM NaCl and 50 mM xanthine; ST+UA, MS with 50 mM NaCl and 20 mM uric acid. [file 12870_2021_2831_MOESM2_ESM.jpg]

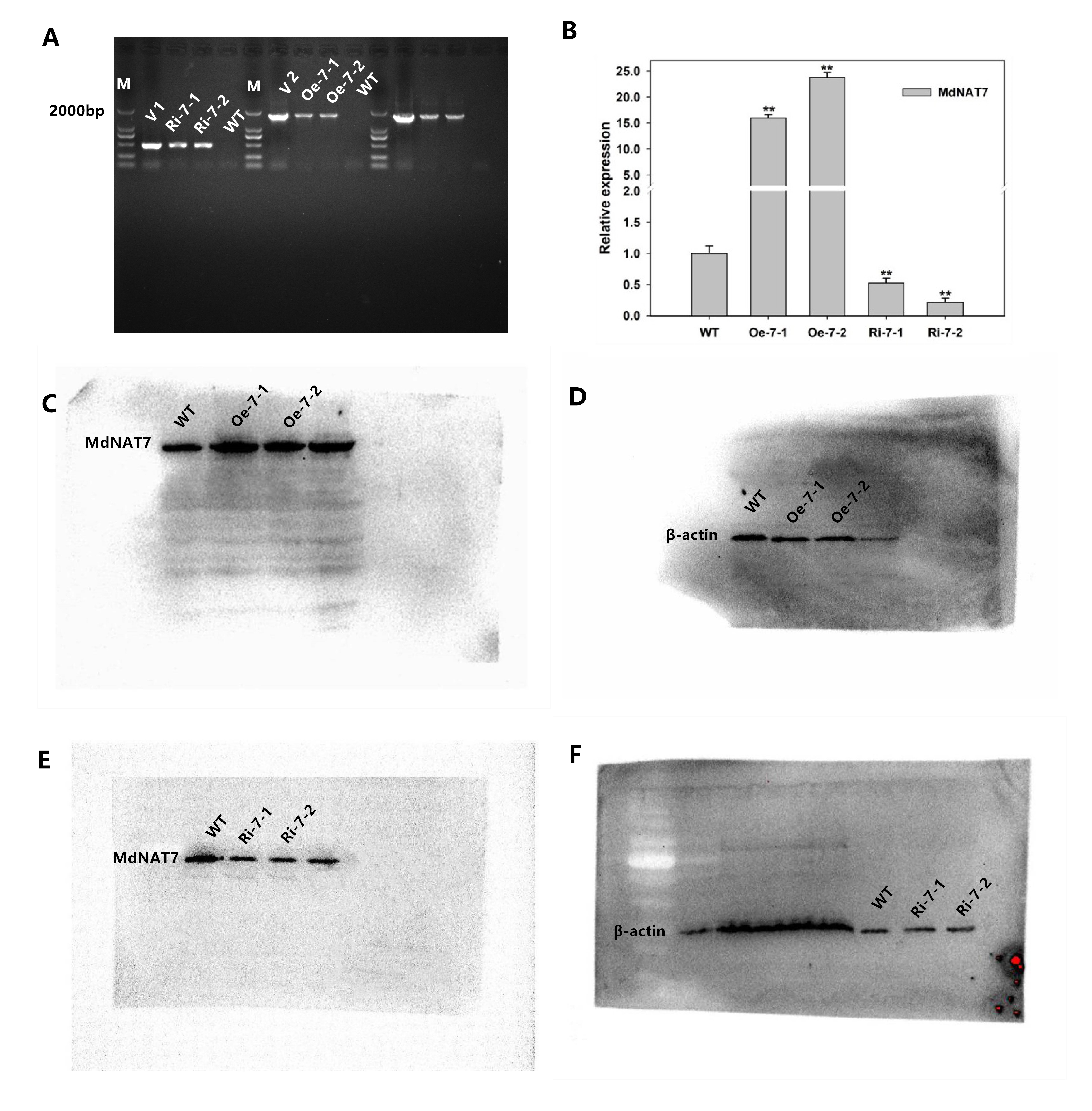

Supplement: Supplementary file 3 — Additional file 3: Fig. S2. PCR, qRT-PCR and western blot analysis of the transgenic apple plants. A. PCR results for Ri and Oe transgenic apple plants detection. M, molecular marker DL2000; V1, positive vector that contains the Hellsgate2-MdNAT7 plasmid; Ri-7-1 and Ri-7-2, MdNAT7-Ri transgenic lines; WT, wild type; V2, positive vector that contains the pCambia121-MdNAT7 plasmid; Oe-7-1 and Oe-7-2, MdNAT7-Oe transgenic lines. B. MdNAT7 expression in Oe and Ri plants by qRT-PCR. C. Western blot analysis of MdNAT7 protein in WT and Oe transgenic apple plants. D. Western blot analysis of β-actin protein in WT and Oe transgenic apple plants. E. Western blot analysis of MdNAT7 protein in WT and Ri transgenic apple plants. F. Western blot analysis of β-actin protein in WT and Ri transgenic apple plants. Apple leaf samples from WT and transgenic apple plants were collected under normal growth conditions. Data are the means and SDs of three replicates. Asterisks indicate significant differences between WT and transgenic lines according to Tukey’s multiple range tests (P < 0.05). [file 12870_2021_2831_MOESM3_ESM.jpg]

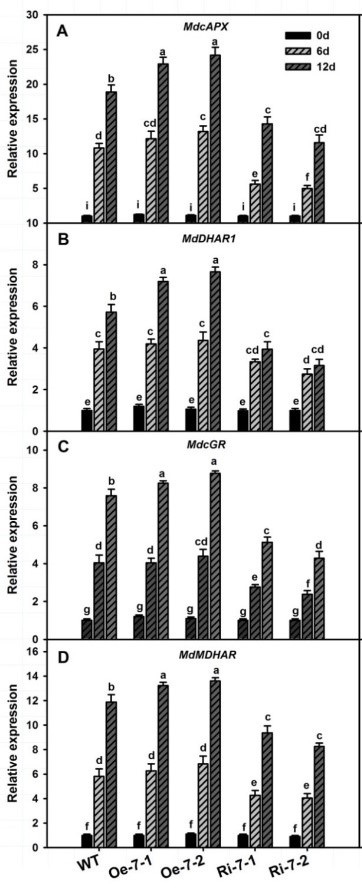

Supplement: Supplementary file 4 — Additional file 4: Fig. S3. Changes in the levels of transcripts for the genes involved in the AsA–GSH cycle during the salt stress period: A–D. MdcAPX, MdDHAR1, MdcGR, and MdMDHAR in plant leaves. Measurements were made at 0, 6 and 12 days of treatment. Data are the means and SDs of three replicates. Different letters indicate significant differences between treatments according to Tukey’s multiple range tests (P < 0.05). [file 12870_2021_2831_MOESM4_ESM.jpg]
